# Seed size and burial depth influence *Zostera marina* L. (eelgrass) seed survival, seedling emergence and initial seedling biomass development

**DOI:** 10.1371/journal.pone.0215157

**Published:** 2019-04-11

**Authors:** Martin Søndergaard Jørgensen, Rodrigo Labouriau, Birgit Olesen

**Affiliations:** 1 Department of Bioscience, Aarhus University, Aarhus, Denmark; 2 Department of Mathematics, Aarhus University, Aarhus, Denmark; Università della Calabria, ITALY

## Abstract

Seed burial in the sediment is critical for successful seedling establishment in seagrasses because it protects from predation and dispersal into unsuitable sites, and it may enhance germination by exposing the seeds to suitable germination stimuli. However, relatively little is known about the fate of buried seeds and their ability to emerge from greater depths. The goal of this study was to determine seed survival in the sediment, seedling emergence success and initial seedling biomass of *Zostera marina* in relation to burial depth and to evaluate if large seeds, having larger energy reserves, are more tolerant to burial than small seeds. Seeds from a perennial *Z*. *marina* population were buried at 7 different sediment depths (0.1–8 cm), and seeds sorted by size (large and small) were buried at depths of 2, 4 and 6 cm in outdoor mesocosms. Total seedling emergence after 2 months was significantly affected by seed burial depth, with maximum values in the top 2 cm of the sediment (48.1–56.7% of planted seeds), and a marked decline below 4 cm depth to only 5% seedling emergence at the deepest burial depth of 8 cm. Moreover, seeds had shorter time to emergence from shallow compared to deep burial depths. At all burial depths, a small fraction of seeds (<10%) died after germination but before emerging, and 15–30% remained viable after 6 months. Seed mortality was the major limitation to seedling recruitment from the deeper burial depths. The effect of seed size on seedling emergence success and time was not clear, but heavier seeds displayed greater longevity and gave rise to seedlings of significantly higher biomass, indicating that the mobilization of metabolic reserves may be important during initial seedling development.

## Introduction

*Zostera marina* (eelgrass) is a widespread and often dominant seagrass species in coastal and estuarine ecosystems across the northern hemisphere, where it fulfils important roles as ecosystem engineer and service provider [1,2]. Widespread seagrass declines have led to increased awareness of the need for conservation and restoration of these vulnerable ecosystems and their capacity to recover from impacts associated with environmental pressures [3–5].

As most clonal plants, *Z*. *marina* can use both asexual and sexual reproduction to recover from disturbances. While clonal growth is the principal mode for sustaining persistent meadows, sexual recruitment plays a vital role in population expansion and recovery, and is necessary for the colonization of areas outside the range of vegetative growth [6–9]. In addition, sexual recruitment influences population connectivity and contributes to increased genetic variation, which may enhance long-term resilience to stress [10].

Sexual recruitment in plant populations is a multi-stage sequential process and is the combined result of seed production and dispersal, and the prevailing environmental conditions at the time of dormancy relief, germination and seedling emergence [11]. For seagrasses, the period between seed deposition on the sediment surface and seedling establishment may last several months, and is often considered a major limitation to successful recruitment [12–14]. Mature *Z*. *marina* seeds are negatively buoyant and settle to the bottom after release from the reproductive shoots. Here some seeds may be lost due to predation, attack by pathogens or dispersal to unfavourable sites, while those that become entrapped in the sediment provide a seed bank, where they may remain dormant and viable for more than 12 months [12,15]. Successful recruitment from seed banks require specific signals to break dormancy and induce germination [16]. For *Z*. *marina*, seed germination is affected by environmental factors such as temperature, oxygen concentration and salinity [17,18], but may also be strongly influenced by burial in the sediment [19–21]. The vertical profile of *Z*. *marina* seed banks is influenced by currents and wave- driven sediment deposition [8] and the feeding and burrowing activities of benthic fauna [22,23]. Hence, viable eelgrass seeds have been found down to sediment depths of 8 cm in an annual seagrass meadow [24] and as deep as 14 cm in a dieback area of a former perennial meadow [25].

Seed burial can be favourable to sexual recruitment, because buried seeds become less accessible to predation [26,27], they are less exposed to removal by water movement, and seedlings may develop root systems with better anchoring capacity, reducing the risk of being dislodged during events of physical stress [28]. Furthermore, anoxic conditions stimulate germination of *Z*. *marina* seeds, and moderate seed burial may enhance germination as compared to germination at the oxygenated sediment surface [17,18]. However, burial below a certain threshold may affect seedling emergence negatively. In deeply buried seeds, the axial hypocotyl must elongate further in order for the cotyledon to reach the sediment surface, after which the first true leaf can develop [29,30]. Hypocotyl elongation is supported by seed starch reserves that are gradually depleted during the germination process [30]. Consequently, the energy demand of germination is expected to increase with increased burial depth, and deeply buried seeds may not have sufficient energy reserves for the seedling to reach the sediment surface. The length of the axial hypocotyl under field conditions can reach up to 53 to 58 mm [25,31], and laboratory experiments have confirmed that seedlings can emerge from depths of 5 to 6 cm [19,20]. A high burial depth may also lead to delayed emergence, thereby prolonging the period during which the seed embryo is exposed to anoxic conditions and toxic end products of anaerobic respiration [32]. This suggests that there is an optimal sediment depth for successful sexual recruitment, and that the vertical distribution of the seed bank is critical for successful seedling establishment. The dependency of seed germination on storage reserves suggests that larger seeds, which contain more starch, may extend the range of burial depths from which successful emergence is possible [33]. In addition, larger starch reserves may enable seeds to persist longer when unfavourable conditions are encountered, as suggested by [19].

In this study, we investigate how burial depth and size of *Z*. *marina* seeds influence seed germination and mortality, seedling emergence and subsequent seedling biomass development in outdoor mesocosms. We hypothesized that seedling emergence success decreases and become delayed with increasing seed burial depth, and that heavier seeds, having larger starch reserves, are able to germinate and emerge from greater depths. With increased seed burial depth or reduced seed size, the relative proportion of starch reserves allocated to the germination process increases, and less energy will remain to support the formation of the first true leaf and early seedling growth. We therefore also hypothesized that the biomass of newly emerged seedlings decrease with increased seed burial depth and reduced seed mass.

## Material and methods

### Seed collection and characteristics

Reproductive shoots were collected from a *Z*. *marina* meadow (1–2 m depth) in Limfjorden, Denmark (57.03 N, 9.41 E) in late June 2014 and placed in flow-through tanks with seawater (salinity ~20). No specific permission was required to sample eelgrass shoots from this area. In mid-August the mature seeds that had settled to the bottom were collected, transferred to mesh bags, and stored in outdoor containers with aerated seawater at in-situ temperatures at Påskehøjgård, Aarhus University (56.22 N, 10.12 E). From March 2015 and until experimental start in late April, the seeds were stored in the laboratory at 5 °C to reduce germination.

To ensure seed viability, seeds that were soft when pinched with a forceps and seeds with split seed coat were discarded from the experimental seed pool. Seed viability was tested on 30 seeds by immersion in a 0.5% tetrazolium chloride (TTC) solution for 24 hours at room temperature, following procedures outlined in [34]. A portion of the seeds was then sorted by eye into batches of large and small seeds. Seed size was measured on 100 seeds with natural size distribution (unsorted seeds hereafter) and on 30 seeds from the batches of large and small seeds (sorted seeds hereafter), respectively. These seeds were scanned (HP Scanjet G4050) and the dimensions of the major (length) and minor (width at widest point) axes of each seed were measured in ImageJ v.1.49. Seed volume was calculated assuming that eelgrass seeds conform to the shape of a prolate ellipsoid [35]. The seeds were then oven dried at 60 °C for 24 h and weighed with 0.001 mg accuracy.

### Sediment characteristics

Sandy sediment was collected from a shallow unvegetated location, and sieved through a 2 mm mesh to remove pebbles and large invertebrates. To estimate sediment organic content, samples dried at 105 °C until constant weight were combusted at 550 °C for 24 hours and weight loss was calculated. Sediment grain size composition was determined by sequential sieving through meshes corresponding to coarse (>0.5 mm), medium (>0.25 mm) and fine sand (>0.063 mm), and silt/clay (<0.063 mm) [36]. The filtered sediment was dried at 105 °C for 24 h and weighed. At the end of the experiment, vertical redox profiles were measured at 1 cm interval in pots containing sediment with no added seeds (see below) using a platinum electrode. The electrode was lowered into the sediment and allowed to equilibrate until the drift was less than 1 mV per minute before a measurement was taken.

### Experimental setup

The experiment was performed in five outdoor tanks (0.9 m in diameter) with 125 L artificial seawater (Marinemix professional, HW Wiegand GmbH) with a salinity of ~20 and continuous water circulation and aeration. Salinity was measured on a weekly basis and adjusted as needed. Water temperature was recorded every 30 min by HOBO temperature loggers (U22-001) placed in each tank at the start of the experiment.

To examine the effect of seed size and burial depth on seed germination, seed mortality, seedling emergence, and seedling biomass unsorted seeds were placed at seven different sediment depths (0.1, 1.0, 2.0, 3.0, 4.0, 6.0, and 8.0 cm), and small and large seeds, respectively, were placed at three different depths (2.0, 4.0, and 6.0 cm). We prepared two depth-series of unsorted seeds to be able to an analyse the fate of seeds remaining in the sediment and the biomass of seedlings, just after the period of maximum seedling emergence (sampled after 2 months) and at the end of the growing season (sampled after 6 months). Pots (11 x 11 x 11 cm) were filled with sediment to target planting depths and placed in seawater at 5 °C for 24 hours, where after additional sediment was added to adjust for compression. Fifteen seeds were distributed evenly in each pot and sediment was added to one cm below the rim of the pots. In addition, ten pots containing sediment with no seeds, were included for analysis of redox profiles. The pots were then transferred to the outdoor tanks with one replicate pot in each of the 5 tanks, yielding a total of 70 pots with unsorted seeds (7 depths, 2 sampling times) and 30 pots (3 depths) with small and large seeds, respectively.

### Seedling emergence, seedling biomass and fate of remaining seeds

Seedling emergence, defined by the emergence of the cotyledon at the sediment surface, was followed from 23 April and until the pots were destructively sampled on 26 June and 15 October for the two series of pots with unsorted seeds, respectively, and on 5 July for pots with large and small seeds. At 3–7 days interval, with decreasing frequency as new seedlings ceased to emerge, new seedling were recorded and marked with identification numbers to follow their fate over time. Following termination of the experiment, all seedlings were carefully removed from the pots, separated into above- and belowground tissue at the point where the first pair of roots protruded, and dried at 60 °C for 24 h. Unemerged seeds were recovered by sieving the sediment through a 0.6 mm mesh and separated into 1) viable seed, which were intact, hard seeds tested for viability with TTC staining, as described above 2) unsuccessfully emerged seeds defined as seed coats split open and with missing embryo, and 3) dead seeds, which were either soft or failed the TTC staining test.

### Statistical analysis

Seed size metrics were analysed with Kruskal-Wallis test, since the data did not satisfy assumptions of homoscedasticity as determined by Levene’s test. Post hoc analyses of the data was performed with Dunn´s test.

The temporal patterns of seedling emergence were analyzed using survival/time-to-event analysis methods. The data consisted of observations of the number of seeds that had emerged at some specified time points, and therefore the exact time at which a seed emerged was unknown, but instead, it was known to have emerged between two consecutive observation times, i.e. the emergence times were interval censored. The survival functions (i.e., the function that associates a given time with the probability of emergence after that time) for the distribution of the emergence times were estimated using a non-parametric estimate specially designed for interval censored times as described in [37] and [38]. The log-rank test was used to test for significant differences between groups [39] using the R-package “interval”. The survival function was integrated using a trapezoidal approximation to obtain estimates of the mean/expected times for emergence, and confidence intervals (with 95% coverage) were obtained using non-parametric bootstrap with 10,000 bootstrap samples.

Total seedling emergence was quantified as the cumulated percentage of emerged seedlings at the end of the experiment. Because seedling emergence ceased after approximately 2 months, we included total seedling emergence from the two depth-series of unsorted seeds sampled in July and October, respectively. Statistical inference for the total seeding emergence from unsorted seeds was made using binomial quasi-likelihood methods (i.e. generalized linear mixed models with the quasi-binomial distribution), since the data presented clear signs of over-dispersion (a large ratio between the deviance and the number of degrees of freedom). The importance of individual model terms and interactions was assessed via a likelihood ratio test. Post hoc comparisons between treatments were performed with pairwise comparisons. Generalized linear models with the quasi-binomial distribution were used to test for the effect of burial depth and sampling time (June and October) and their interaction on fate of unemerged seeds (viable, dead and unsuccessfully emerged seeds, respectively), and for the effect of seed size (large and small seeds) and burial depth and their interaction on seedling emergence and fate of unemerged seeds.

The effect of seed size and burial depth on seedling above- and belowground biomass was analysed with one-way ANOVA for unsorted seeds and two-way ANOVA for sorted seeds. Normality and homoscedasticity of the data were tested with Shapiro-Wilk’s and Levene’s test.

All of the statistical analysis were computed in in RStudio [40].

## Results

### Seed material

Initial seed viability, tested with TTC staining, was high (98.4% ± 1.6 (SE)). The mean size of unsorted, small, and large seed size classes differed significantly with regards to width, volume and dry weight, while the length of seeds from the unsorted and small seed size classes were similar (Table 1). The median seed mass was 3.6 mg DW for unsorted seeds and 2.9 and 4.9 mg DW for the small-sized and large-sized seed classes, respectively. There was a slight overlap in seed weight distribution between the small and large seeds, ranging from 1.4–3.9 and 3.2–7.0 mg DW, respectively, while the weight distribution of unsorted seeds ranged from 1.4–6.6 mg DW, spanning the entire range of seed weights in the two seed size classes.

**Table 1:** Size metrics of the three *Z*. *marina* seed size classes used in the experiment. Values are median with 95% confidence intervals. Letters indicate significant difference (P<0.05) between the seed size classes.

	Length (mm)	Diameter (mm)	Volume (mm^3^)	Weight (mg DW)
**Unsorted seeds**	3.45 (3.42–3.54)^a^	1.61 (1.59–1.67)^b^	4.72 (4.52–5.03)^b^	3.55 (3.15–3.75)^b^
**Large seeds**	3.70 (3.64–0.80)^b^	1.88 (1.82–1.94)^c^	6.70 (6.47–7.29)^c^	4.91 (4.54–5.12)^c^
**Small seeds**	3.43 (3.39–3.48)^a^	1.53 (1.51–1.58)^a^	4.32 (3.98–4.46)^a^	2.92 (2.54–3.22)^a^

### Sediment characteristics and water temperature

The sediment had a low organic content (0.29% DW ± 0.003), and was composed primarily of coarse sand (68.5% DW ± 0.1) and only a small fraction of silt and clay (0.3% DW ± 0.1). The sediment redox potential was significantly affected by depth (ANOVA; p< 0.001), and decreased to anoxic levels (Eh < 0) at depths below 3 cm, where it remained at a constant level of around -50 mV down to 8 cm depth. Water temperature increased from 16 °C to 19 °C during the first three weeks of the experiment, when most of seedlings emerged. This was followed by a short cooling period until mid-May, where after temperature gradually increased from 13°C to 21°C until mid-august, and then decreased again to 12°C in mid-October when the experiment was finalized.

### Seedling emergence

Seedlings emerged from all seed burial depths from 0.1 to 8 cm and with a nearly synchronously appearance across all depths during the third week of the experiment (not shown). Hence, a large proportion of the total seedling emergence (82.7%) occurred in one week, and gradually ceased during the following five weeks. In spite of this synchronicity, significant differences between burial depths were detected, with delayed emergence time for unsorted seeds buried deeper than 4 cm (Table 2). Hence, the expected day of emergence increased from 39.6–42.2 days for seeds buried at 0.1 to 3 cm to 55.3 days at 6 cm (Table 2). Due to small sample size, the emergence time of seeds from 8 cm was excluded from the analysis. For sorted seeds, the emergence time was not significantly affected by seed size or by seed burial depth although the expected day of emergence tended to increase with increasing seed burial depth (Table 2).

**Table 2:** Expected time to 50% seedling emergence of unsorted, large and small seed size classes. Letters indicate significant difference (P<0.05) between seed sediment depths of unsorted seeds and between seed size and sediment depth of the small and large seed size classes.

Depth (cm)	Expected day of emergence (95% CI)		
	Unsorted seeds	Large seeds	Small seeds
**0.1**	39.6 (37.2–42.15)^a^	-	-
**1**	40.6 (38.7–42.4)^a^	-	-
**2**	40.0 (38.0–41.9)^a^	32.8 (30.9–34.6)^a^	33.30 (31.4–35.3)^a^
**3**	42.2 (39.9–44.6)^ab^	-	-
**4**	45.3 (42.8–47.7)^b^	35.1 (31.8–37.4)^a^	34.25 (32.3–36.0)^a^
**6**	55.3 (51.4–60.2)^c^	36.40 (33.9–38.7)^a^	37.81 (34.7–40.6)^a^

**Fig 1:**
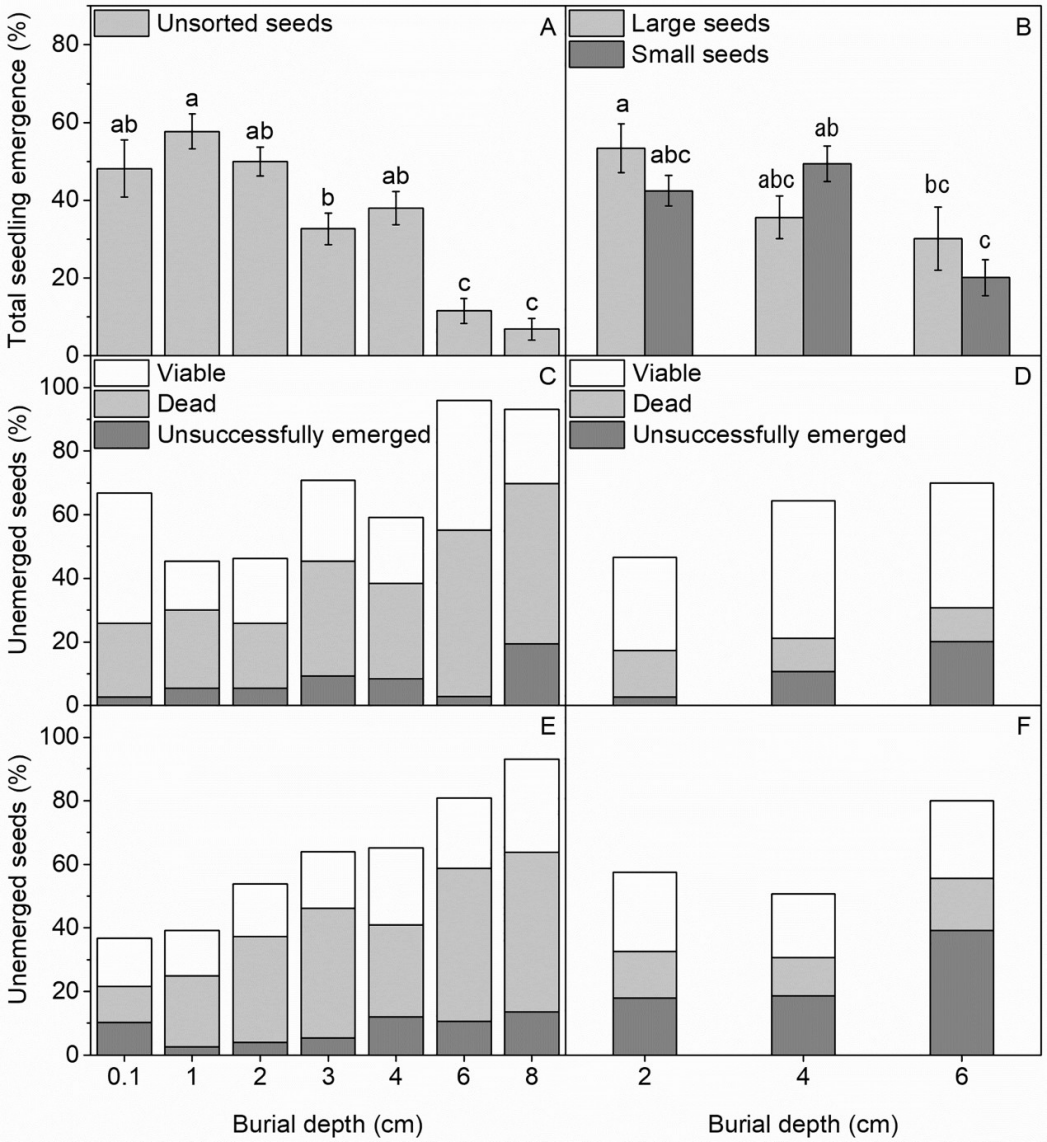
Seedling emergence and remaining seeds at sediment burial depths. Mean percentage of total emerged seedlings from unsorted (A) and sorted (B) seeds 2 months after planting at different sediment depths. Letters indicate significant difference (P<0.05) between depths. Mean percentage of total planted seeds that were viable, dead or unsuccessfully emerged after 2 months (C) and 6 months (E) for unsorted seeds, and after 2.5 months for large (D) and small (F) seeds, at the different seed burial depths.

Total seedling emergence was significantly affected by seed burial depth for both sorted and unsorted seeds (Fig 1A and 1B, Table 3). For unsorted seeds, the total seedling emergence was greatest for seeds buried at 0.1–2 cm (48.1–56.7%) and decreased significantly to 12 and 6.2% for seeds buried at depths of 6 and 8 cm, respectively (Fig 1A). Likewise, burial depth affected the total seedling emergence of sorted seeds, but there was no significant difference between large and small seeds (Fig 1B, Table 3). Overall, total seedling emergence was lowest from 6 cm sediment depth for both large (29.3%) and small seeds (20.0%), but large seeds showed highest emergence from 2 cm depth (53.3%) and small seeds from 4 cm (49.3%), as indicated by the significant interaction between burial depth and seed size (Fig 1B, Table 3).

**Table 3:** Likelihood ratio tests for the effect of burial depth on total seedling emergence. Generalized linear mixed model testing the effect of sediment depth on seedling emergence from unsorted seeds, and generalized linear model for effect of depth and seed size on emergence from large and small seed size classes (sorted seeds).

	Parameter	d.f.	Dev.	Res. d.f.	Res. Dev.	P-value
**Unsorted seeds**	Null model			8	430.85	
	Depth	6	164.8	2	266.05	**<0.001**
**Sorted seeds**	Null model			29	51.93	
	Seed size (A)	1	0.17	28	51.76	0.680
	Depth (B)	2	18.34	26	33.42	**<0.001**
	A x B	2	6.69	24	26.73	**0.035**

### Remaining, unemerged seeds

The fraction of unsorted seeds that germinated but did not successfully emerge, as indicated from the number of empty, split seed coats, was unaffected by seed burial depth and time after planting (2 and 6 months), and constituted a small fraction of 8.0 ± 1.3% of total planted seeds across all seed burial depths (Fig 1C and 1E, Table 4). The fraction of dead seeds was unaffected by time of sampling, but differed significantly between burial depths and was much higher at 6 to 8 cm depth (50.2%) than at 0.1 and 1 cm (17.1 and 23.4%, respectively) (Fig 1C and 1E, Table 4). The fraction of seeds that remained viable in the sediment was significantly affected by seed burial depth, sampling time and their interaction (Table 4). Hence, the fraction of viable seeds decreased from June to October, particularly for seeds buried at 0.1 (from 41.2 to 15.3%) and 6 cm (from 40.7 to 22.2%) (Fig 1C and 1E). Overall, the fraction of viable seeds tended to increase with depth and varied at the end of the six months experimental period from 14.5% at 1 cm burial depth to 29.5% at 8 cm (Fig 1E).

**Table 4:** Likelihood ratio test for effect of burial depth on unemerged seeds remaining in the sediment. Generalized linear model (GLM) testing the 1) effect of seed sampling time (June and October) and burial depth on the unemerged, unsorted seeds that were dead, viable or had failed to emerge and 2) the effect of seed size and burial depth on unemerged seeds from the planted large and small seed size classes.

		Parameter	d.f.	Dev.	Res. d.f.	Res. Dev.	P-value
**Unsorted seeds**	Unsuccessfully emerged	Null model			69	160.18	
		Time (A)	1	0.19	68	160.05	0.803
		Depth (B)	6	19.12	62	140.93	0.157
		A x B	6	13.05	56	127.88	0.385
	Dead	Null model			69	170.89	
		Time (A)	1	0.24	68	170.66	0.708
		Depth (B)	6	62.73	62	107.92	**<0.001**
		A x B	6	7.82	56	100.10	0.592
	Viable	Null model			69	78.43	
		Time (A)	1	5.94	68	72.49	**<0.001**
		Depth (B)	6	18.14	62	54.34	**<0.001**
		A x B	6	10.92	56	43.43	**0.020**
**Sorted seeds**	Unsuccessfully emerged	Null model			29	69.58	
		Seed size (A)	1	15.15	28	54.43	**<0.001**
		Depth (B)	2	20.96	26	33.48	**<0.001**
		A x B	2	2.87	24	30.60	0.307
	Dead	Null model			29	55.89	
		Seed size (A)	1	0.54	28	55.35	0.613
		Depth (B)	2	0.84	26	54.50	0.820
		A x B	2	0.45	24	54.05	0.899
	Viable	Null model			29	41.39	
		Seed size (A)	1	11.24	28	30.16	**0.001**
		Depth (B)	2	1.19	26	28.96	0.583
		A x B	2	3.01	24	25.95	0.256

Seed size had a significant effect on both the fraction of seeds that failed to emerged and seeds that remained viable at the conclusion of the 2.5 month experiment, whereas the fraction of dead seeds was low irrespective of seed size and burial depth (overall average: 13.2%) (Fig 1D and 1F, Table 4). The fraction of unsuccessfully emerged seeds varied significantly between seed burial depths and with seed size, and constituted a higher proportion of small (17.9 to 39.1%) than large (2.7 to 20.1%) seeds (Fig 1D and 1F, Table 4), and showed and overall increase with planting depth. The fraction of unemerged seeds that remained viable was significantly affected by seed size, and constituted an overall higher fraction for large (37.2 ± 1.8%) than small (23.1 ± 2.7%) seeds (Fig 1D and 1F, Table 4).

### Seedling biomass

**Fig 2:**
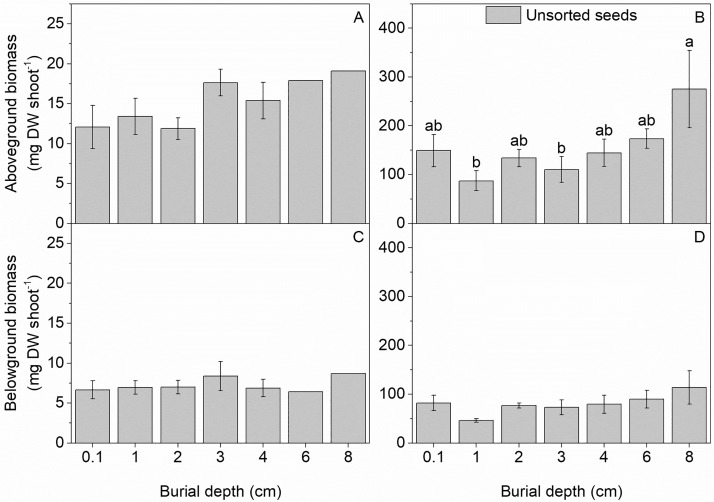
Effect of sediment depth on biomass of *Z*. *marina* seedlings of unsorted seed origin. Mean above- (A, B) and belowground (C, D) biomass of seedlings originating from unsorted seeds and harvested in June and October, 2 and 6 months after experimental start, respectively. Values are mean ± SE.

The above- and belowground biomass of seedlings that originated from unsorted seeds increased nearly 10-fold during the 4 months between first and second sampling (Fig 2), whereas the above- to belowground biomass ratio only changed slightly from 2.1 ± 0.1 to 1.9 ± 0.1 (not shown). While the biomass of seedlings was unaffected by seed burial depth at the first sampling in June, 4–5 weeks after peak seedling emergence, the aboveground biomass was significantly higher for seedlings originating from deeply buried seeds (8 cm: 275.5 mg DW seedling^-1^) compared to shallow seeds (1 cm: 87.4 mg DW seedling^-1^) 4 months later (Fig 2, Table 5).

**Table 5:** ANOVA test results for effects of burial depth on seedling biomass. One-way ANOVA testing the effect of seed burial depth on the biomass (mg DW seedling^-1^) of seedlings emerging from unsorted seeds two (June) and six (October) months after planting, respectively, and two-way ANOVA testing the effect of seed burial depth and seed size on seedlings emerging from small and large seed-size classes (sorted seeds).

	Variable	Parameter	d.f.	F-value	P-value
**Unsorted seeds—June**	Aboveground biomass	Depth	6	1.30	0.300
			21		
	Belowground biomass	Depth	6	0.31	0.925
			21		
**Unsorted seeds–Oct.**	Aboveground biomass	Depth	6	3.03	**0.022**
			26		
	Belowground biomass	Depth	6	1.38	0.261
			26		
**Sorted seeds**	Aboveground biomass	Depth (A)	2	0.56	0.577
		Seed size (B)	1	7.13	**0.013**
		A x B	2	0.28	0.762
			24		
	Belowground biomass	Depth (A)	2	0.13	0.875
		Seed size (B)	1	9.67	**0.005**
		A x B	2	0.11	0.895
			24		

**Fig 3:**
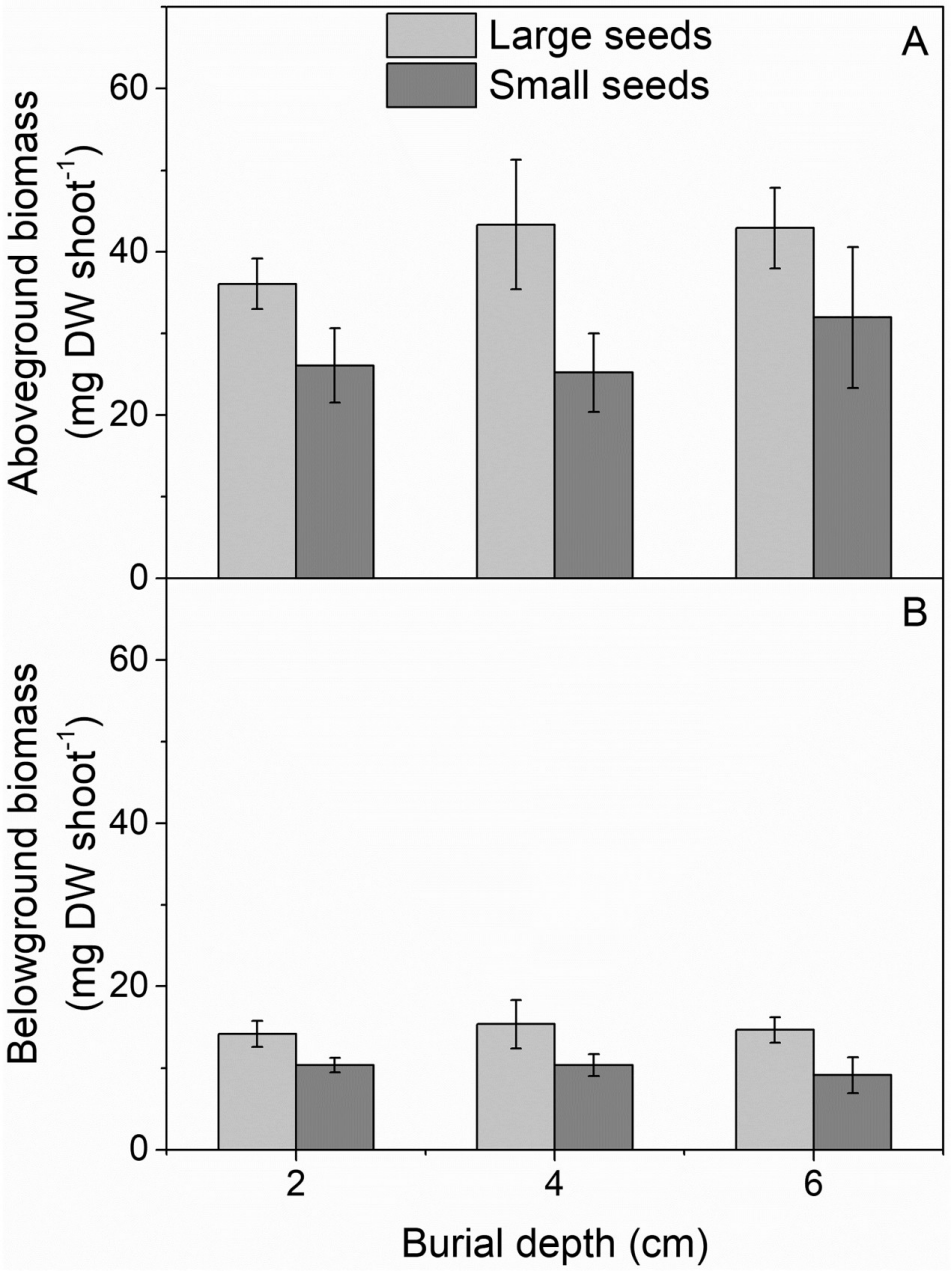
Biomass of *Z*. *marina* seedlings originating from large and small seeds. Mean aboveground (A) and belowground biomass (B) of seedlings harvested in July, 2.5 months after experimental start. Values are mean ± SE.

The biomass of seedlings derived from large and small seeds was not significantly affected by seed burial depth at time of sampling (5–6 weeks after peak emergence), but large seeds gave rise to significantly larger seedlings that on average weighed 1.5 times more than seedlings formed by small seeds (Fig 3, Table 5).

## Discussion

### Critical depth for successful seedling emergence

In this study, seed germination leading to successful seedling emergence occurred at sediment depths down to 8 cm, but increasing seed burial depth severely reduced the number of emerging seedlings and delayed the time of emergence from sediment depths below 3 cm. Seedling emergence tended to be greatest within the top 2 cm, which corresponds to the optimal range of sediments depths to maximize seedling emergence previously identified for *Z*. *marina* [20,21].

The maximum depth from which seeds can recruit probably depends on sediment properties such as grain size, which may impose physical constraints on hypocotyl elongation [19,20,41]. However, comparable studies of burial limits for *Z*. *marina* seeds suggest threshold sediment depths of 5–6 cm [20]. This is similar to the maximum sediment depth in natural populations as estimated from the hypocotyl length measurements (5.3–5.8 cm) of emerged seedlings [25,31]. Hypocotyl elongation of emerging seedlings varies directly with seed germination depth [29,32], but their length may slightly underestimate the potential seed burial depth. Hence, measurements of subsurface seedling lengths in this study showed that hypocotyl elongation from seeds buried below 2 cm depth stopped when the base of the cotyledonary sheath reached a sediment depth of 1.7–2.3 cm (Jørgensen, unpublished data). This enables subsequent formation of adventitious roots to take place at a position in the sediment that may ensure firm anchorage during the early stage of seedling development [28].

Low seedling emergence from deeply buried seeds can be due to pre-emergence mortality of germinated seeds or absence of germination, either because the microenvironment at depth lacks the required cues to stimulate dormancy relief or germination, or because it affects seed mortality. Pre-emergence seedling mortality has for terrestrial plant species been linked to insufficient seed energy reserves to support seedling elongation until reaching the soil surface and transition to autotrophy [42]. Similar effect of seed depth on emergence failure has been suggested in a study of *Z*. *marina* seed bank dynamics, where the proportion of empty, split seed coats, indicating unsuccessful emergence, was 4 times higher at deep (5 cm) compared to shallow (1 cm) sediment depths [19]. In this study, the presence of unsuccessfully emerged seeds was generally low and unaffected by depth, although the proportions tended to be higher at 4–8 cm depth (12–13.6%) than at 1–3 cm depth (2.7–5.3%) after 6 months in the sediment. Instead, the low seedling emergence from greater depths was caused by increased seed mortality as well as decreased germinability, as indicated by the increase in the number of viable seeds with depth. The major loss of seeds due to mortality occurred within the first 2 months of the experiment, and with the fraction of dead seeds increasing more than 2-fold from 2 to 8 cm depth. Seeds below 3 cm depth experienced anoxic conditions (Eh < 0) and below 4 cm the redox values were consistently negative (-50 mV), and it is likely that exposure to reduced metabolites lead to increasing seed mortality with depth. While exposure to hypoxic and anoxic conditions may stimulate *Z*. *marina* seed germination [17,18], lethal effects of organic enriched sediments and low redox conditions have been described for *Z*. *marina* seeds [43,44] and propagules of freshwater macrophytes [45]. However, it is not clear whether this was caused by accumulation of reduced elements such as ammonium or sulphide or soluble organic toxins derived from anaerobic microbial activity [46]. Ammonium and sulphide can be toxic for aquatic macrophytes even at micromolar concentrations in the water phase [47,48]. However, the moderate redox potentials measured in this study indicate low sulphide concentrations, and sulphide exposure was probably of minor importance to seed mortality. It is possible that the pre-experimental conditions of cold stratification (2 months at 5 °C) influenced the dormancy state at the time of planting. This may have increased the germinability of the seeds upon transfer to experimental conditions, thereby activating embryo metabolism leading to reduced seed persistence to the chemical environment at depth. However, to fully understand the effects seed burial depth on seed mortality, interactive effects of redox correlates and the state of seed dormancy need to be further investigated.

### Seed size effect on germination and seedling emergence

The rate and percentage of seedling emergence with increased seed burial depth did not strictly reflect seed size. Hence, the proposed advantage of larger carbohydrate reserves in heavy seeds did not result in higher seedling emergence success than for seeds with smaller energy reserves. In general, large seeds show higher percentage seedling emergence and are more likely to emerge from greater depths than small seeds [42,49], but the opposite has also been observed [50]. For *Z*. *marina*, the relatively fast elongation of the hypocotyl proceeds mainly through cell expansion and accumulation of water rather than by cell division [29], which may partly explain the similar length growth and time of emergence for large and small seeds in this study. However, a much higher percentage of small compared to large seeds died after germinating but before emerging, as indicated from the number of split seed coats. This suggests that smaller seeds were more receptive to environmental stimuli than larger seed, but had a reduced ability to reach the surface from greater burial depths. Hence, earlier loss of dormancy in small seeds of terrestrial plants species has been attributed to larger surface to volume ratio as well as thinner seed coats, allowing for more rapid uptake of water [50]. In contrast, the number of unemerged seeds that remained viable in the sediment throughout the study period was much higher among large *Z*. *marina* seeds suggesting longer persistence than for small seeds. Similar results were also reported by [15], who showed that large *Z*. *marina* seeds had greater survival than smaller seeds during long-term storage in water cultures. The causes of delayed germination of large seeds are unknown, but may be related to seed characteristics such as thicker seed coats that excludes environmental cues and protects the embryo against adverse pore water conditions. However, the increased inhibition of germination with depth suggests that interactive effects of sediment properties related to depth were involved. The higher persistence of large seeds may provide an ecological advantage in broadening the period over which seed germination and seedling emergence can occur, and by enhancing potential seed recruitment following events of sediment movement, e.g. through erosion or bioturbation that bring the seeds closer to the sediment surface.

### Effects of seed burial depth and size on seedling biomass

Contrary to our hypotheses, the initial seedling biomass was not negatively affected by germination depth, although the emergence from deeply buried seeds (4–6 cm) was delayed by 5–15 days compared shallower seeds, and by the end of the six month experimental period, seedlings originating from deeply buried seeds even tended to have larger biomass.

Positive effects of burial on early seedling growth and establishment have previously been described for *Z*. *marina* [19] and could result from a deeper positioned hypocotyl, increasing the access to sediment nutrient reserves. However, our data on seedling biomass could also be biased due to depth dependent selection, if the few seeds that managed to emerge from large depth were those with larger energy reserves or higher metabolic activity.

Regardless of seed burial depth, large seeds gave rise to seedlings that in the early stages of development produced more biomass than seedlings originating from smaller seeds. This suggests that large-seeded seedlings benefit from a greater amount of metabolic reserves when emerging from the sediment surface, because a larger fraction of the seed carbon and nutrient reserves may be unconsumed. Other studies have shown that seedling biomass increases with seed mass, and that this may result in higher survival and competitive ability during early seedling establishment [49–51]. However, differences in seedling size are often most evident at time of emergence and may not persist over time [42,49]. Hence, the greater biomass of large-seeded eelgrass seedlings may be of greatest significance to their initial establishment by improving survival under stressful conditions such as shade, sediment instability or nutrient limitation.

## Conclusions

Like most seagrass species, *Z*. *marina* is regularly found at highly disturbed sites, where sediment dynamics caused by hydrodynamic forces or activity of burrowing animals may influence adult plant performance and re-establishment from seeds [12,28,52,53]. In this study, experimental burial of seeds indicated that seedling recruitment from *Z*. *marina* seed banks may occur from a sediment depth of 8 cm, which is substantially deeper than observed for seeds of other submerged angiosperm species (1–3 cm) [41,54]. However, only a small fraction of seeds below 4 cm depth emerged successfully, and increased seed mortality suggests that a part of the seed bank may be lost permanently due to burial. The optimum depth for successful recruitment was within the top 2 cm, which is in accordance with previous studies [20,21]. While moderate burial generally is considered advantageous for seedling recruitment, seeds located on the sediment surface germinate slower [17] and are more vulnerable to losses caused by export to unfavourable sites, uprooting due to poor anchorage [28] and predation [26,27].

*Z*. *marina* seed banks provide a means for re-establishment of degraded meadows [7,25,55], and the vertical distribution of seeds in the sediment should be considered when predicting potential recovery from the seed bank. In contrast to our predictions, the rate and percentage of seedling emergence from depth was unaffected by seed size. However, a much larger fraction of large seeds remained viable, while more small seeds were lost from the seed bank due to failed emergence. Moreover, large seeds produced larger seedlings than small seeds. This suggests that the pronounced variation in seed size observed within and among eelgrass populations [33,35] may significantly affect the probability of seed to seedling transition and initial seedling establishment.

## Supporting information

S1 Table Raw data of seed size metrics. (XLSX)

S2 Table Raw data of seedling emergence. (XLSX)

S3 Table Raw data of unemerged seeds. (XLSX)

S4 Table Raw data of seedling biomass. (XLSX)
